# Risk factors of postoperative nausea and vomiting following ambulatory surgery: A retrospective case-control study^☆^

**DOI:** 10.1016/j.heliyon.2022.e12430

**Published:** 2022-12-19

**Authors:** Yue Qian, Jian-kun Zhu, Bai-ling Hou, Yu-e Sun, Xiao-ping Gu, Zheng-liang Ma

**Affiliations:** a Department of Anesthesiology, Nanjing Drum Tower Hospital, Clinical College of Nanjing Medical University, Nanjing, China; b Department of Anesthesiology, Nanjing Drum Tower Hospital, The Affiliated Hospital of Nanjing University Medical School, Nanjing, China

**Keywords:** Postoperative nausea and vomiting, Ambulatory surgery, Postoperative pain

## Abstract

**Objective:**

To explore potential risk factors of postoperative nausea and vomiting (PONV) following ambulatory surgery.

**Method:**

Clinical data of 1670 cases receiving ambulatory surgery in Nanjing Drum Tower Hospital, the Affiliated Hospital of Nanjing University Medical School from September 2017 to December 2019 were retrospectively analyzed. They were categorized to PONV group and non-PONV group, and perioperative data in both groups were analyzed for assessing risk factors of PONV following ambulatory laparoscopy.

**Results:**

There were 156/1,670 (9.3%) PONV cases, and the female and male incidence in recruited cases was 12.0% and 6.0%, respectively. Analyses on perioperative data of them identified that female gender [adjusted odds ratio (aOR) = 2.060, *P* < 0.001], operation time >1 h (aOR = 1.554, *P* = 0.011), postoperative pain at rest (aOR = 1.909, *P* = 0.013) and postoperative pain during activities (aOR = 3.512, *P* < 0.001) were independent risk factors of PONV following ambulatory surgery. Furthermore, postoperative pain at rest and during activities were linearly, positively correlated to the incidence of PONV.

**Conclusion:**

Female gender, operation time >1 h and postoperative pain are closely related with the incidence of PONV following ambulatory surgery. Alleviating postoperative pain properly is one of the methods to reduce risk factors of PONV following ambulatory surgery.

## 1 Introduction

Ambulatory surgery is a medical mode in which admission, surgery or procedure (excluding outpatient surgery) and discharge are completed within 24 h, which was initially proposed by Dr. Nicholl in England [1]. The concept of the Enhanced Recovery after Surgery (ERAS) has further accelerated the development of ambulatory surgery, which highlights the optimization of perioperative approaches to prevent perioperative stress and inflammatory response, and thus improve the safety and comport of patients based on evidence-based medicine [2, 3]. The aim of ambulatory surgery is to achieve rapid recovery and elevate physical and psychological comforts of patients.

Postoperative nausea and vomiting (PONV) is a common postoperative complication in patients with general anesthesia. Its incidence is about 20%–30% [4], which is as high as 70%–80% in high-risk patients. PONV not only causes a strong subjective discomfort in patients, but results in complications like disturbance of water and electrolyte balance, acid-base imbalance, wound dehiscence, or aspiration pneumonia in severe cases. As a consequence, PONV may increase medical cost, length of stay and the incidence of re-admission [5, 6, 7]. The incidence of PONV in patients receiving ambulatory surgery is not lower than that of elective surgery, which is usually underestimated because of a quick postoperative observation and evaluation. It is reported that the incidence of PONV following ambulatory surgery is about 10.8% [8], which achieves 25% in patients receiving laparoscopic surgery [9, 10]. Through literature review, potential influencing factors of PONV in Chinese population have been rarely reported. This study intends to retrospectively analyze risk factors of PONV in Chinese patients receiving ambulatory surgery.

## 2 Method

### 2.1 Recruitment of participants

A total of 1,670 patients receiving ambulatory surgery in Nanjing Drum Tower Hospital, Clinical College of Nanjing Medical University from September 2017 to December 2019 were recruited. Participants with incomplete clinical data or quitted ambulatory surgery were excluded. This study was approved by the Ethic Committee of Nanjing Drum Tower Hospital and was registered in the Chinese Clinical Trials Registry.

### 2.2 Baseline data collection

Preoperative, intraoperative, and postoperative data of participants were collected as follows: (1) preoperative data were collected from the electronic medical record system, including age, gender, comorbidities, cerebral infarction, carsickness, and history of smoking and drinking. (2) Intraoperative data were collected from the electronic anesthesia system, including type of anesthesia, type of operation, operation time, intraoperative medication, analgesia method and ASA class. (3) Postoperative data were collected from the follow-up system, including PONV and VAS score. Nausea and vomiting occurred after recovery from anesthesia were recorded as PONV. All data were directly extracted by the information center, and the extracted personnel have no knowledge of the content of this experiment.

### 2.3 Statistical analyses

SPSS 20.0 was used for data processing. Measurement data were expressed as $\bar{x}$ ± s, and compared using the independent Student's *t* test. Enumeration data were expressed as percentage (%), and compared using Chi-square test or Fisher's exact test. Potential influencing factors screened out from the univariable Logistic regression model were later introduced in the multivariable Logistic regression model, aiming to identify independent risk factors of PONV. For the independent variables with serious multicollinearity problems, the stepwise regression method was used to automatically eliminate the collinearity independent variables. Receiver operating characteristic (ROC) curves were depicted for assessing the ability of the model to distinguish PONV cases from patients receiving ambulatory surgery through calculating area under the curve (AUC). Hosmer-Lemeshow goodness-of-fit test was performed to assess the calibration of the estimated model. The correlation between VAS score and the incidence of PONV in patients receiving ambulatory surgery was evaluated by Chi-square test. A significant difference was set at *P* < 0.05.

## 3 Results

### 3.1 Baseline characteristics of participants

A total of 156/1,670 (9.3%) patients receiving ambulatory surgery developed PONV, including 112 (12.0%) females and 44 (6.0%) males. They were categorized to PONV group (n = 156) and non-PONV group (n = 1 514). Baseline characteristics of them in both groups were listed in Table 1. A significantly higher rate of female patients, longer operation time, higher rate of laparoscopic surgery patients and higher incidence of postoperative pain were observed in PONV group than those of non-PONV group (all *P* < 0.05). No significant differences in the type of anesthesia and length of stay were identified between groups (*P* > 0.05).

**Table 1:** Baseline characteristics of participants in PONV and non-PONV group.

Factors	PONV group n = 156	non-PONV group n = 1,514	χ^2^	*P*
Age (years)			2.130	0.144
0-60 (n, %)	132 (84.6%)	1207 (79.7%)		
>60 (n, %)	24 (15.6%)	307 (20.3%)		
Gender			16.499	<0.001
Female (n, %)	112 (71.8%)	825 (54.5%)		
Male (n, %)	44 (28.2%)	689 (45.5%)		
ASA class			0.076	0.783
Ⅰ-Ⅱ (n, %)	149 (95.5%)	1453 (96.0%)		
III (n, %)	7 (4.5%)	61 (4.0%)		
Comorbidities				
Hypertension (n, %)	6 (3.8%)	80 (5.3%)	0.599	0.439
Diabetes (n, %)	3 (1.9%)	18 (1.2%)	0.614	0.438
Cardiac diseases (n, %)	0 (0.0%)	30 (2.0%)	2.086	0.248
Lung diseases (n, %)	2 (1.3%)	8 (0.5%)	1.35	0.238
Renal insufficiency (n, %)	1 (0.6%)	3 (0.2%)	1.161	0.325
History of cerebral infarction (n, %)	0 (0.0%)	5 (0.3%)	0.517	1.000
History of smoking and drinking (n, %)	0 (0.0%)	2 (0.1%)	0.206	1.000
History of carsickness (n, %)	0 (0.0%)	2 (0.1%)	0.206	1.000
Type of operation			4.658	0.031
Laparoscopy (n, %)	118 (75.6%)	1017 (67.2%)		
Non-laparoscopy (n, %)	38 (24.4%)	497 (32.8%)		
Operation time			5.502	0.019
≤1 h (n, %)	81 (51.9%)	932 (61.6%)		
>1 h (n, %)	75 (48.1%)	582 (38.4%)		
Type of anesthesia				
General intravenous anesthesia (n, %)	135 (86.5%)	1336 (88.2%)	0.392	0.532
Intravenous anesthesia (n, %)	2 (1.3%)	43 (2.8%)	1.309	0.431
Balance anesthesia (n, %)	17 (10.9%)	121 (8.0%)	1.575	0.210
General anesthesia + nerve block (n, %)	2 (1.3%)	8 (0.5%)	1.35	0.238
Nerve block (n, %)	0 (0.0%)	4 (0.3%)	0.413	1.000
Local anesthesia (n, %)	0 (0.0%)	2 (0.1%)	0.206	1.000
Intraoperative medication				
Inhalation anesthetics (n, %)	17 (10.9%)	121 (8.0%)	1.575	0.210
Dexmedetomidine (n, %)	46 (29.5%)	426 (28.1%)	0.127	0.721
Antiemetics			9.534	0.023
Not used (n, %)	5 (3.2%)	157 (10.4%)		
Single drug medication (n, %)	75 (48.1%)	699 (46.2%)		
Dual drug combination (n, %)	66 (42.3%)	542 (35.8%)		
Triple drug combination (n, %)	10 (6.4%)	116 (7.7%)		
Postoperative analgesia				
Opioids (n, %)	12 (7.7%)	69 (4.6%)	3.012	0.113
NASIDs (n, %)	140 (89.7%)	1293 (85.4%)	2.188	0.139
Others (n, %)	3 (1.9%)	10 (0.7%)	2.919	0.114
Postoperative pain				
At rest (n, %)	125 (80.1%)	771 (50.9%)	48.505	<0.001
During activities (n, %)	142 (91.0%)	963 (63.6%)	47.497	<0.001
Postoperative complications				
Delayed discharge (n, %)	2 (1.3%)	9 (0.6%)	1.022	0.312
Dizziness and headache (n, %)	1 (0.6%)	7 (0.5%)	0.095	0.758

PONV, postoperative nausea and vomiting; ASA, American Society of Anesthesiologists; NASIDs, non-steroidal anti-inflammatory drugs.

### 3.2 Risk factors of PONV

Potential influencing factors screened out from the univariable Logistic regression model were later introduced in the multivariable Logistic regression model. The data showed that female gender (aOR = 2.060, *P* < 0.001), operation time >1 h (aOR = 1.554, *P* = 0.011), postoperative pain at rest (aOR = 1.909, *P* = 0.013) and postoperative pain during activities (aOR = 3.512, *P* < 0.001) were independent risk factors of PONV following ambulatory surgery (Table 2).

**Table 2:** Analyses on risk factors influencing PONV following ambulatory surgery.

Factors	Regression coefficient	χ^2^	*P*	OR (95% CI)
Female gender	0.732	14.721	<0.001	2.060 (1.424, 2.979)
Operation time				
≤1 h	–	–	–	(Control)
>1 h	0.441	6.494	0.011	1.554 (1.107, 2.182)
Postoperative pain at rest	0.647	6.178	0.013	1.909 (1.147, 3.180)
Postoperative pain during activities	1.256	12.492	<0.001	3.512 (1.175, 7.048)

PONV, postoperative nausea and vomiting; OR, odds ratio; CI, confidence interval.

**Figure 1:**
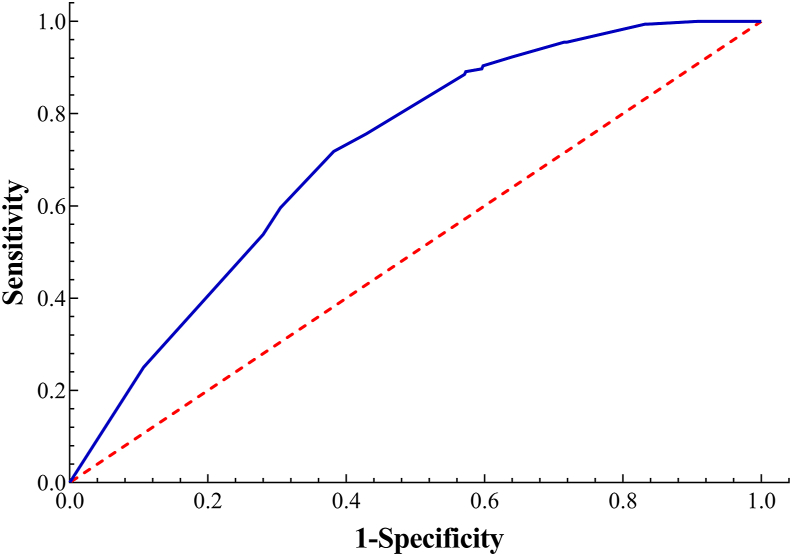
ROC curves depicting the discrimination of the model in distinguishing PONV cases from patients receiving ambulatory surgery.

**Figure 2:**
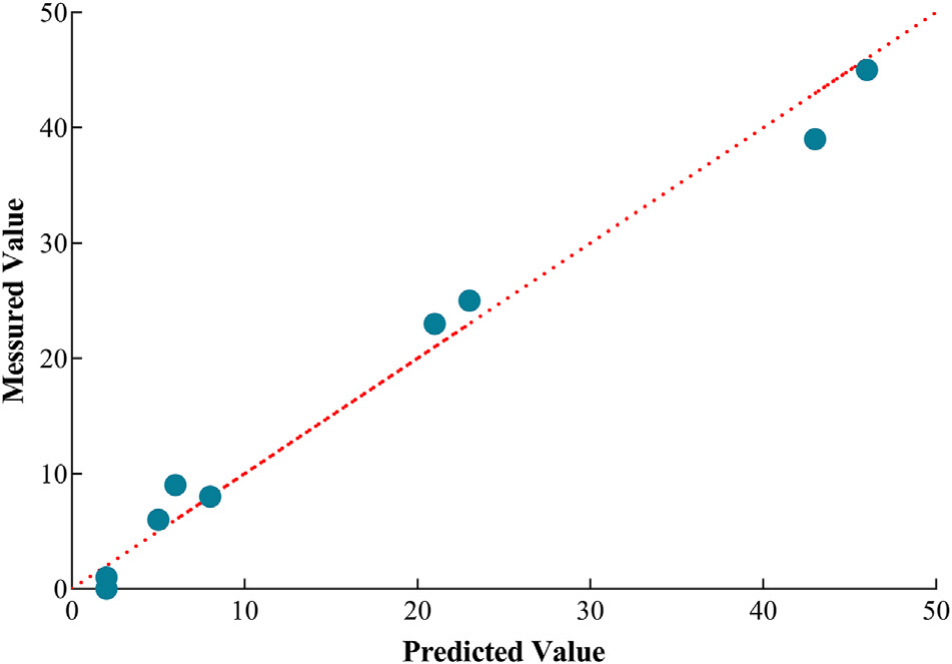
Hosmer-Lemeshow goodness-of-fit test verifying the calibration of the estimated model in distinguishing PONV cases from patients receiving ambulatory surgery.

Furthermore, ROC curves demonstrated that the model was capable of distinguishing PONV cases from patients receiving ambulatory surgery (AUC = 0.712, Figure 1). In addition, Hosmer-Lemeshow goodness-of-fit test verified the calibration of the estimated model with a prediction accuracy of 90.7% (χ^2^ = 5.878, *P* = 0.554) (Figure 2).

### 3.3 Influences of postoperative pain on PONV in patients receiving ambulatory surgery

**Figure 3:**
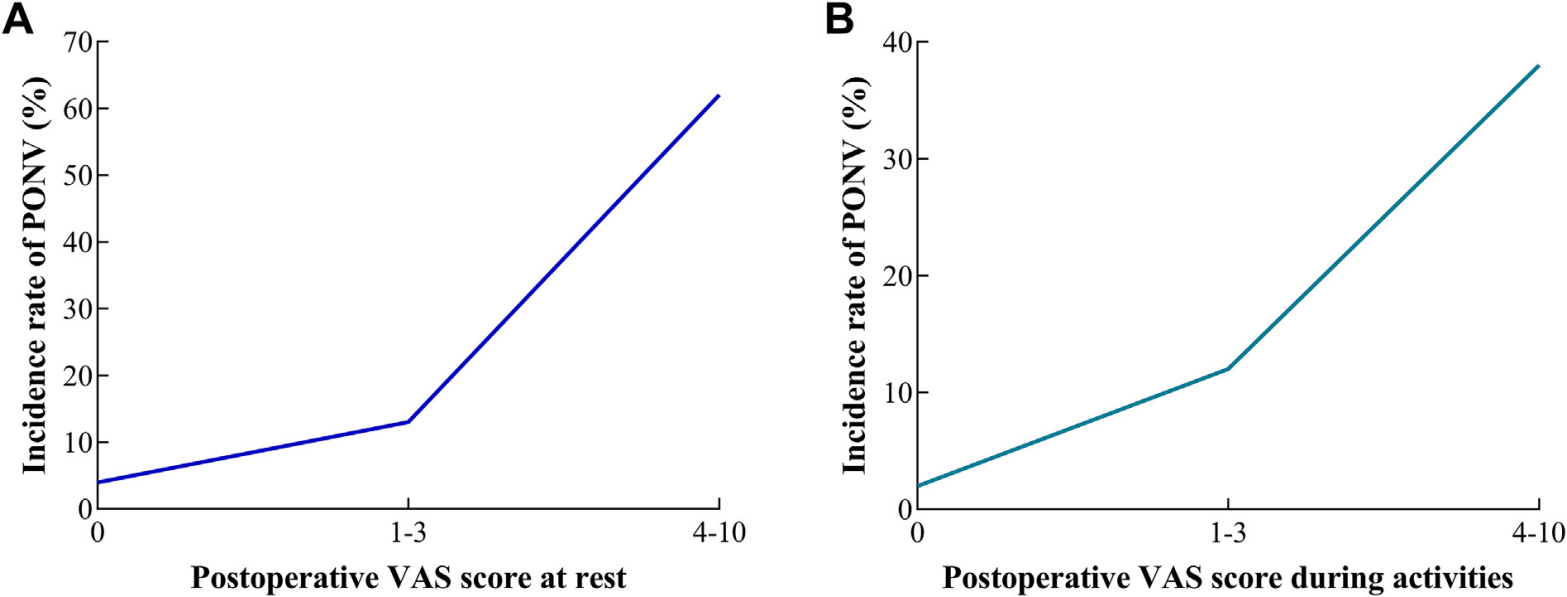
Correlation between postoperative VAS score at rest (A) and during activities (B) and the incidence of PONV following ambulatory surgery.

Chi-square test data revealed that PONV was positively correlated to postoperative pain at rest (χ^2^ = 62.0267, *P* < 0.0001) and during activities (χ^2^ = 70.0971, *P* < 0.0001). It is suggested that the increased incidence of PONV was parallel to the severity of postoperative pain (Figure 3, Table 3).

**Table 3:** Chi-square analysis on the correlation between postoperative VAS score and the incidence of PONV following ambulatory surgery.

Factors	PONV group n = 156	non-PONV group n = 1,514	χ^2^	*P*
VAS at rest			62.0267	<0.0001
0 (n, %)	31 (19.9%)	743 (49.1%)		
1-3 (n, %)	117 (75.0%)	766 (50.6%)		
4-10 (n, %)	8 (5.1%)	5 (0.3%)		
VAS during activities			70.0971	<0.0001
0 (n, %)	14 (9.0%)	551 (36.4%)		
1-3 (n, %)	125 (80.1%)	935 (61.8%)		
4-10 (n, %)	17 (10.9%)	28 (1.8%)		

VAS, visual analogue scale; PONV, postoperative nausea and vomiting.

## 4 Discussion

At present, the most simple and reliable way to prevent PONV is multimodal prevention. Apfel simple risk score [11] is a commonly used preoperative evaluation strategy. Its evaluation factors included: women, non-smokers, PONV or motion sickness history, and postoperative opioids. The study confirmed that the use of simple risk score for hierarchical assessment and prevention can significantly reduce the incidence of PONV [12]. However, some studies believe that the factors affecting the incidence of PONV may not be limited to the above four items. After using the risk score, about 10% of patients undergoing ambulatory surgery still have PONV [13]. It can be seen that the existing PONV prediction model cannot perfectly predict the occurrence of PONV and guide clinical preventive medication.

The effect of gender on PONV has been confirmed by many experiments. A study on elderly patients pointed out that women are one of the main independent risk factors for PONV, which coincides with the results of this study. Phillips et al. [13] reported that although female gender is a vital influencing factor of PONV, it does not have a significant correlation with the incidence of PONV. Kocaturk and Bourdaud et al. [14, 15] also found that gender was not an applicable risk factor when assessing PONV in children. The results of each experiment are different due to the difference of the population, age distribution, operation type and anesthesia method. Therefore, some studies have proposed that although women are an important factor affecting PONV, there is not necessarily related to PONV [13].

A retrospectively analysis reported that laparoscopic surgery and operation time are independent predictive factors of high-risk PONV [16]. Surgical factors that confer increased risk of PONV mainly include surgical stimulations like artificial pneumoperitoneum and traction response, and anesthetics [17, 18]. In particular, opioids directly target the μ-opioid receptors in the chemoreceptor trigger zone, and the reflex vomiting center thereby causes vomiting [19]. The longer duration of operation significantly increases surgical and anesthetic stresses, and the incidence of PONV. Our results showed that operation time >1 h markedly increased the incidence of PONV in patients receiving ambulatory surgery, whilst laparoscopic surgery was not correlated to the incidence of PONV. It may be attributed to the application of multi-mode approaches of preventing PONV in most of laparoscopic surgery patients.

Evidences have demonstrated that the use of volatile anesthetics can cause PONV by directly interacting with neuronal ion channel protein subunits [20]. The longer the patient is exposed to inhalation anesthetics, the incidence of PONV is higher. Our previous study identically found that the use of sevoflurane during gastrointestinal surgery in elderly patients is a risk factor of PONV [21]. However, the effect of sevoflurane on PONV was not found to be significantly different in this study. Here, only 1/141 patients using inhalation anesthetics did not use antiemetics, and there were 32, 65 and 43 cases treated with single, dual and triple drug combination of antiemetics, respectively. This graded anti PONV therapy has been proven to significantly reduce the incidence of PONV after inhalation anesthesia [22], which may be the reason why this study did not yield differential results. Considering the small sample size of this study, the study on the correlation between inhaled anesthetics and PONV may need to increase the sample size in the future or use prospective experimental methods for in-depth verification.

We assessed the influence of postoperative pain on PONV by grading VAS score. It is found that postoperative pain (VAS > 3) was positively correlated to the incidence of PONV in patients receiving ambulatory surgery. The pain center and vomiting center are both located in the thalamus. The stimulation of a certain brain nucleus induces an interaction between nuclei, thus further stimulating other nuclei. As a result, severe pain would induce PONV as well. Meanwhile, the use of high doses of analgesics, especially opioids, is also an important cause of PONV [19]. In this study, since the ambulatory surgical anesthesia in our hospital adopts a standardized anesthesia mode, and the types and doses of opioids used in the operation are relatively fixed, the relationship between opioids and PONV has not been analyzed in this study, which is also the limitation of this study and needs further research in the future.

Collectively, our study identified that female gender, operation time >1 h, postoperative pain at rest and postoperative pain during activities are independent risk factors of PONV following ambulatory surgery. Multi-mode approaches of preventing PONV and analgesia (especially the use of opioids) are recommended for reducing the incidence of PONV in patients receiving ambulatory surgery.

## Declarations

### Author contribution statement

Yue Qian, MD: Performed the experiments; Analyzed and interpreted the data; Wrote the paper.

Jian-kun Zhu, MB: Performed the experiments; Analyzed and interpreted the data.

Bai-ling Hou, MD: Performed the experiments.

Yu-e Sun, MD: Contributed reagents, materials, analysis tools or data.

Xiao-ping Gu, MD; Zhengliang Ma: Conceived and designed the experiments.

### Funding statement

Professor Zhengliang Ma was supported by Jiangsu Commission of Health [Jiangsu Provincial Key Medical Discipline], 10.13039/501100001809National Natural Science Foundation of China [81971044].

This work was supported by 10.13039/501100001809National Natural Science Foundation of China [81771142].

### Data availability statement

Data will be made available on request.

### Declaration of interests statement

The authors declare no conflict of interest.

### Additional information

No additional information is available for this paper.
